# Anti-thymocyte globulin (ATG) differentially depletes naïve and memory T cells and permits memory-type regulatory T cells in nonobese diabetic mice

**DOI:** 10.1186/1471-2172-13-70

**Published:** 2012-12-14

**Authors:** Chang-Qing Xia, Anna V Chernatynskaya, Clive H Wasserfall, Suigui Wan, Benjamin M Looney, Scott Eisenbeis, John Williams, Michael J Clare-Salzler, Mark A Atkinson

**Affiliations:** 1 Department of Hematology, Xuanwu Hospital, Capital Medical University, #45 Changchun Street, Xicheng District, Beijing, P.R. China; 2 Department of Pathology, Immunology and Laboratory Medicine, University of Florida College of Medicine, Gainesville, FL 32610, USA; 3 Genzyme Corporation, Framingham, MA, 02142, USA

**Keywords:** Anti-thymocyte globulin, Naïve and memory T cells, Regulatory T cells, T helper cell, Autoimmune diabetes, Nonobese diabetic mouse

## Abstract

**Background:**

ATG has been employed to deplete T cells in several immune-mediated conditions. However, whether ATG administration affects naïve and memory T cell differently is largely unknown.

**The context and purpose of the study:**

In this study, we assessed how murine ATG therapy affected T cell subsets in NOD mice, based on their regulatory and naïve or memory phenotype, as well as its influence on antigen-specific immune responses.

**Results:**

Peripheral blood CD4+ and CD8+ T cells post-ATG therapy declined to their lowest levels at day 3, while CD4+ T cells returned to normal levels more rapidly than CD8+ T cells. ATG therapy failed to eliminate antigen-primed T cells. CD4+ T cell responses post-ATG therapy skewed to T helper type 2 (Th2) and possibly IL-10-producing T regulatory type 1 (Tr1) cells. Intriguingly, Foxp3+ regulatory T cells (Tregs) were less sensitive to ATG depletion and remained at higher levels following in vivo recovery compared to controls. Of note, the frequency of Foxp3+ Tregs with memory T cell phenotype was significantly increased in ATG-treated animals.

**Conclusion:**

ATG therapy may modulate antigen-specific immune responses through inducing memory-like regulatory T cells as well as other protective T cells such as Th2 and IL-10-producing Tr1 cells.

## Background

Anti-thymocyte globulin (ATG), trade name thymoglobulin®, has been employed for decades as an immune modulator for a variety of clinical indications. It is currently one of the most common immunosuppressive reagents used in allogeneic transplantation [1-3] and more recently, in the treatment of a variety of autoimmune disorders [4-8]. There is a common belief that ATG therapy functions through complement mediated depletion of mature T cells. However, recent data suggests that ATG therapy induces immune modulation beyond that of simple T cell depletion [9]. For example, ATG therapy may facilitate tolerance induction through modulation of dendritic cells (DC), both phenotypically and functionally [10]. Evidence has also shown that ATG therapy may also induce regulatory T cells (Tregs) in vivo [11-13]. However, it remains unclear how ATG therapy affects naive and memory T cells in autoimmune settings such as T1D, although a recent study suggested that ATG therapy effectively eliminates alloantigen specific memory T cells in an allogeneic transplantation mouse model [13]. In the current study, we used both standard NOD mice, as well as a TCR transgenic form of these mice (i.e., NOD.BDC2.5) to investigate changes within immune cell subsets in peripheral blood, spleen and lymph nodes post-ATG therapy, specifically focusing on addressing the questions of how ATG therapy affected naive and memory T cells, including naïve and memory Tregs. These strains were utilized due to their common utilization in studies of murine ATG efficacy for type 1 diabetes, as well as the ability to utilize mice having a defined antigenic specificity. The results demonstrated that ATG therapy differentially depletes T cells from peripheral blood and lymphoid organs. ATG therapy was more efficient in depleting naïve T cells than memory T cells. Tregs appeared resistant to ATG depletion and their frequency remained at increased levels after homeostatic recovery from ATG therapy. It was also noted that proportionately Tregs with memory T cell phenotype were significantly increased post-ATG therapy. Taken collectively, we believe this is a previously unrecognized mechanism whereby ATG therapy differentially affects naïve and memory Tregs.

## Methods

### Mice

Female NOD/Ltj were purchased from Jackson Laboratory and housed in specific pathogen free facilities at University of Florida Animal Care Service. The Institutional Animal Care and Use Committee at University of Florida approved all animal procedures (approval ID: 20090279).

### Media and reagents

RPMI1640 media with glutamine were purchased from Fisher Scientific (Pittsburgh, PA). Complete culture media were prepared using RPMI1640 plus 10% fetal bovine serum (Thermo Scientific, Waltham, MA) and 1x penicillin and streptomycin (Cellgro, Manassas, VA). Murine ATG was provided by Genzyme (Framingham, MA). Rabbit IgG isotype was purchased from the Jackson Laboratory. The following antibodies were purchased from BD Biosciences: (San Jose, CA): CD4-PerCp (clone RM4-5), CD8-FITC (clone 53–6.7), B220-APC (clone RA3-6B2), CD44-APC (clone IM7), CD11c-APC (clone HL3), CD3-PE (clone 17A2) and CD25-APC (clone PC61). The antibodies of CD62L-APC and –FITC (mEL-14) and CD11b-PE (m1/70) were purchased from eBioscience (San Diego, CA). Gr1 (Ly6G/Ly6C)-APC (clone RB6-8C5) and Foxp3-PE, -FITC (clone MF-14) were purchased from BioLegend (San Diego, CA). CellTrace CFSE kits, and CD3, CD28 antibody-coated Dynabeads were purchased from Invitrogen (Carlsbad, CA). Multiplex bead cytokine assay kits were purchased from Millipore (Billerica, MA). Mouse CD11c beads were purchased from Miltenyi Biotech (Germany). CD4+ T cell negative selection kits were purchased from StemCell Biotechnology Inc. (Vancouver, Canada). KLH was purchased from CalbioChem (San Diego, CA). Bovine serum albumin was purchased from Sigma-Aldrich (St. Louis, MO). Alum adjuvant was purchased from Thermo Scientific (Waltham, MA).

### ATG treatment and observation of peripheral blood cell components

6–8 week old NOD mice were treated with two intraperitoneal injections of ATG or isotype IgG (500 ug/mouse), 3 days apart, as previously described [11,12]. In some experiments, two groups of mice were monitored longitudinally by examining white blood cell lineages using flow cytometry (LSRFortessa, BD) including CD4+, CD8+ T cells, B220+ B cells, Gr-1+ granulocytes as well as CD62L+ naive T cells and CD62-CD44+ memory T cells. The data were analyzed by FCS express De Novo software version 3 (Vancouver, Canada).

### Measurement of splenic memory and naive T cells and T cell response to in vitro stimulation post ATG therapy

NOD mice were treated with ATG and isotype IgG as described above. At day 3 or 22, the treated mice were sacrificed. Single cell suspensions of spleen cells were prepared and CD4+, CD8+ T cells as well as CD62L+ naive and CD62-CD44+ memory T cells were examined by flow cytometry (LSRFortessa, BD). A portion of the spleen cells were used for CD4+ T cell isolation using CD4+ T cell negative isolation EasySep kits following the manufacturer’s instructions (StemCell Inc. Canada). Spleen cells (1 × 10^6^) were stimulated with anti-CD3 antibody (3 ug/ml) and in some experiments, purified CD4+ T cells were stimulated with CD3 and CD28 antibody-coated Dynabeads mouse T cell activator (Invitrogen), with splenic DC plus antigens (KLH), or autoantigens (NIT-1 cell lysates) as indicated for 3–4 days, then, ^3^H-thymidine (1 uCi/well) was added to each well for the final 16 hours. Incorporation of ^3^H-thymidine was measured by scintillation counting (Wallac Trilux).

### Measurement of Foxp3+ Treg cells

Spleen cells were stained with anti-CD4-PerCp and anti-CD25-APC. The cells were then fixed and permeablized and stained with anti-Foxp3-PE following the instructions of the manufacturer (eBioscience). CD4+CD25+Foxp3+ Treg cells were examined by flow cytometry (LSRFortessa, BD) and analyzed by FCS express De Novo Software version 3.

### Splenic CD4+ T cell and CD11c+ DC isolation

Spleen cells were freshly prepared. Purified CD4+ T cells were prepared using EasySep CD4+ T cell negative section kit (StemCell Technologies Inc, Vancouver, Canada), and splenic DCs were labeled with anti-CD11c-microbeads, and then isolated by magnetic cell sorting following manufacturer’s instructions (Miltenyi). The purity of CD4+ T cells and CD11c+ DC was approximately 95%.

### KLH immunization and recall response assay

NOD mice were treated with ATG or isotype IgG as described above along with simultaneous immunization by intraperitoneal injections of KLH (25 ug/mouse) in adjuvant Alum. The treated mice were sacrificed at day 22 post-ATG therapy and harvested cells were cultured with stimulators as indicated, or medium only for 4 days. Supernatants (50 ul/well) were harvested and stored for later cytokine assay. Then, ^3^H-thymidine (1 uCi/well) was added to the cultures and incubated for additional 16 hours. ^3^H-thymidine incorporation was measured as described above.

### Autoantigen immunization and recall response in vitro and in vivo

NIT1 cells (NOD insulinoma cell line) were cultured according to the method provided from the vendor (ATCC). NIT1 cells (2 × 10^7^) were suspended into 1 ml PBS. The cell suspension underwent freeze-thaw procedures 4 times to prepare NIT1 cell lysates. NOD mice (6 weeks old) were treated with ATG or isotype IgG as described above along with intraperitoneal injections of 50 μl of NIT1 lysates in 50 μl of Alum.

#### In vitro antigen recall response assay

A week following the last treatment, all mice were sacrificed and spleen cells prepared and stimulated with NIT1 lysates (10 ul/well), a control antigen KLH (10 ug/ml), or medium for 4 days. Supernatants (50 ul/well) were harvested and stored for later cytokine assay. Then, ^3^H-thymidine (1 uCi/well) was added to the cultures and incubated for additional 16 hours. ^3^H-thymidine incorporation was measured by scintillation counting. A portion of the spleen cells from the above mice were used for CD4+ T cell isolation. The isolated CD4+ T cells (2 × 10^5^/well) were stimulated with splenic dendritic cells (2 × 10^4^/well) purified from naive NOD mice in the presence of NIT1 lysates (20 μl/well). The T cell proliferation was measured by ^3^H-thymidine incorporation assay, as described above.

#### In vivo antigen recall response assay

In these experiments, we stained a portion of spleen cells prepared above with carboxyfluorescein succinimidyl ester (CFSE) following the instructions from the manufacturer (Invitrogen). Then, we adoptively transferred CFSE-labeled spleen cells (2 × 10^7^/mouse), as prepared above, into 8-week-old NOD mice. Four days later, the mice were sacrificed and pancreatic lymph node cells, as well as cells from inguinal lymph node, were prepared and the CFSE-labeled T cell proliferation (dilution of CFSE) was examined by flow cytometry. In these experiments, we chose to use CFSE-labeled whole spleen cells but not purified CD4+ T cells because the whole spleen cells would be more reflecting the T cell behaviors post-ATG therapy.

### Cytokine assay

Cytokine concentrations in culture supernatants, including IFN-γ, IL-4, IL-5 and IL-10, were measured by multiplex cytokine assay kits using Luminex 100 (Luminex Map Technology) following the instruction from the manufacturer (Millipore).

### Statistical analysis

Data were analyzed using Student *t* testing. Differences with p<0.05 were considered to be statistically significant.

## Results

### ATG therapy efficiently depletes T cells from peripheral blood, but is less efficient in depleting T cells from lymphoid organs

**Figure 1:**
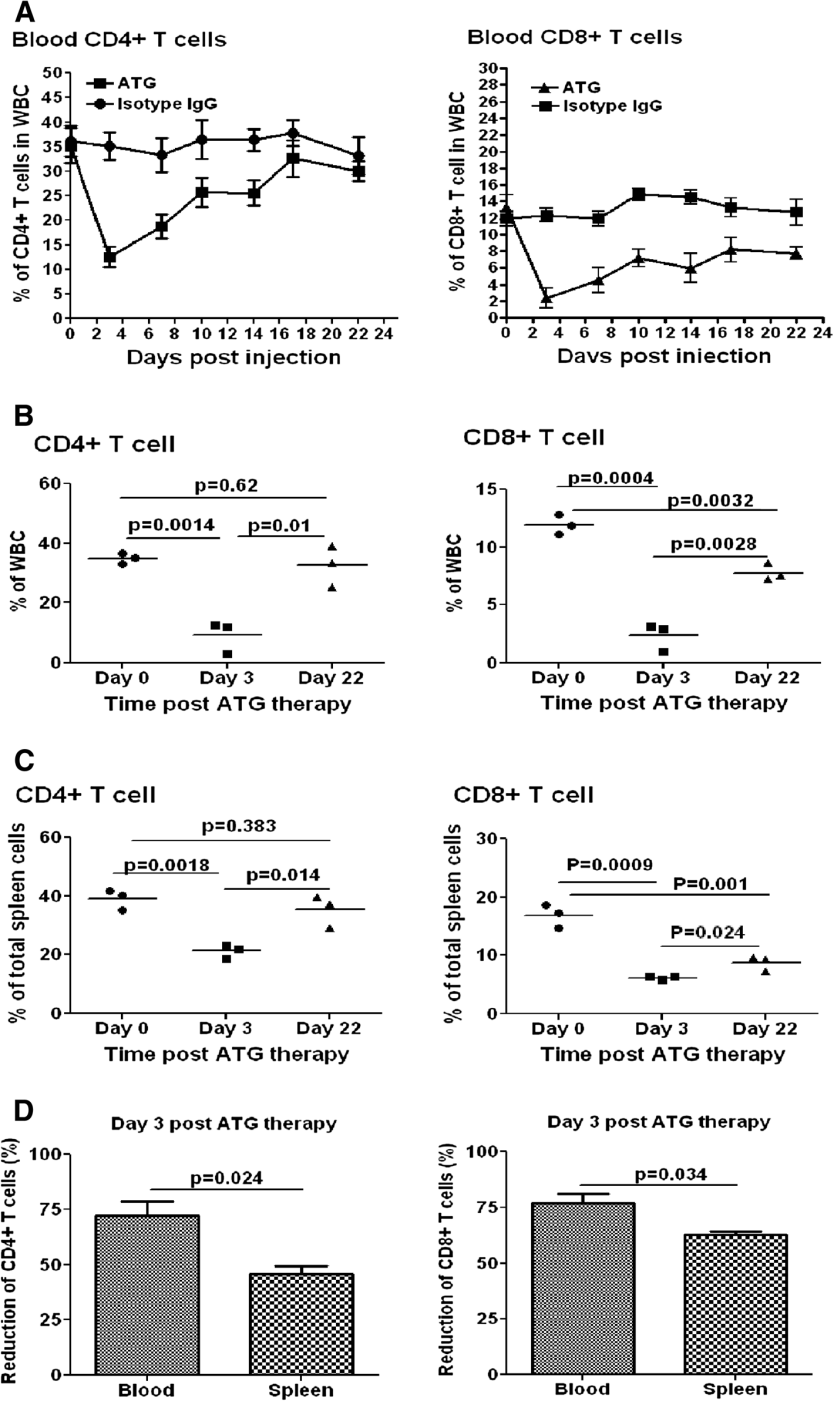
**ATG therapy differentially depletes T cells from peripheral blood and lymphoid organs.** NOD mice were treated with ATG or isotype IgG twice with a 3-day interval. Then, CD4+ and CD8+T cells in peripheral blood were examined by flow cytometry every 3 days until day 22. **A **shows CD4+ and CD8+ T cell percentages in total peripheral white blood cells at different time points post ATG therapy (n=4 mice in each group); **B** and **C **show CD4+ and CD8+ T cell percentages in peripheral white blood cells and spleen cells, respectively (n=3 mice in each group). **D **exhibits CD4+ and CD8+ T cell depletion rates in peripheral blood and spleen at day 3 post ATG therapy, respectively (n=3 mice in each group). The similar results were obtained in additional two independent experiments.

It is known that ATG therapy can largely eliminate T cells from peripheral blood. However, it was of interest to learn to what extent ATG eliminated T cells from lymphoid organs. Our kinetic observation of peripheral blood cells post-ATG therapy revealed that both the CD4+ and CD8+ T cells dropped to their lowest levels at day 3 post-ATG therapy and by day 22, peripheral blood CD4+ T cells returned to normal levels. In contrast, whereas CD8+ T cells were shown to recover, they remained significantly lower than at baseline by day 22 (Figure 1A and Additional file 1: Figure S1). Based on the kinetic changes of blood T cells above, in subsequent experiments, we compared CD4+ and CD8+ T cells in peripheral blood and spleen at day 3 and day 22 post-ATG therapy. Again, we found that at day 3 post-ATG therapy, both CD4+ and CD8+ T cells were drastically reduced in peripheral blood (Figure 1B and D). In contrast, the reduction of both T cell populations in spleen at day 3 post-ATG therapy was significantly less than in peripheral blood (Figure 1C and D). We did not find significant differences between ATG and isotype IgG treated animals in terms of the spleen size and the total cell numbers in spleen at day 3 post-ATG therapy (data not shown and Additional file 1: Figure S2). Therefore, the percentage change would reflect the absolute number change in splenic T cells. Again, by day 22 post-treatment, the percentage of CD4+ T cells did not show significant differences between the ATG group and isotype IgG group, in both blood and spleen (Figures 1B, C and Additional file 1: Figure S1). However, CD8+ T cells were significantly lower in the ATG group than in control animals, in both blood and spleen (Figure 1B, C and Additional file 1: Figure S1). These results indicate that T cell depletion predominantly takes place in peripheral blood and that CD4+ T cells recover faster than CD8+ T cells.

### ATG therapy differentially depletes naive and memory T cells from the peripheral blood and spleen

**Figure 2:**
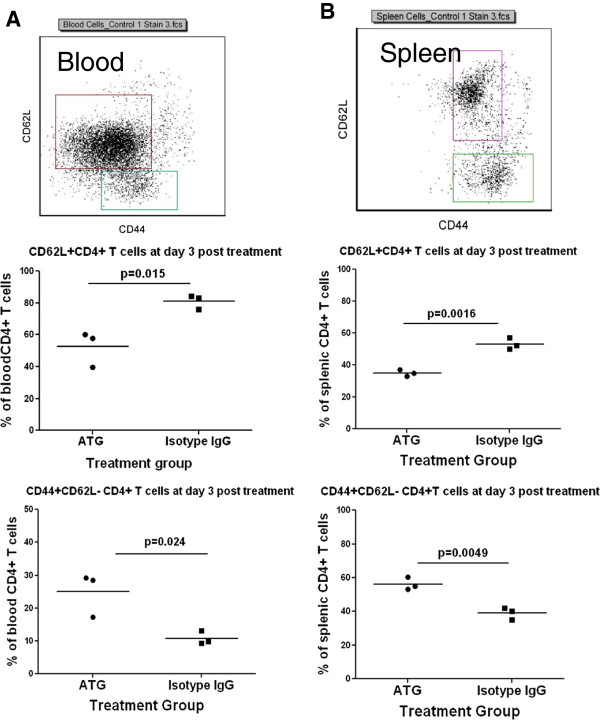
**ATG therapy differentially depletes naive and memory T cells in peripheral blood and spleen. **NOD mice were treated with ATG or isotype IgG twice with a 3-day interval. At day 3 post treatment, CD62L+CD4+ and CD44+CD62L-CD4+ T cells in peripheral blood and spleen were examined by flow cytometry and analyzed by gating CD4+ T cells as depicted in the upper panels of **A **and **B**, respectively. **A **shows the summary of the percentages of CD62L+CD4+ T and CD44+CD62L- CD4+ T cells in total CD4+ T cells in the peripheral blood, respectively (lower panels). **B **shows the summary of the percentages of above T cell subsets in total CD4+ T cells in spleen cells, respectively (lower panels). Three mice were included in each group. Three independent experiments were performed with similar results.

It is still a matter of debate whether ATG therapy preferentially depletes certain subsets of T cells [4,14-17]. In these experiments, we investigated changes of naive and memory T cells using CD62L and CD44 markers (shown in Figure 2), respectively, in peripheral blood and spleen, at day 3 and day 22 post-ATG treatment. We discovered that CD62L+CD4+ (Figures 2A and B) and CD62L+CD8+ naive T cells (data not shown) were significantly reduced at day 3 post-ATG therapy, in contrast to the Isotype IgG group, in both peripheral blood and spleen. Of interest, the percentage of CD62L-CD44+ CD4+ T cells (Figure 2A and B) and CD62L-CD44+CD8+ T cells (data not shown) in total CD4+ and CD8+ T cells, respectively, were significantly increased in the ATG group compared to the control group. These results suggest that ATG therapy may preferentially deplete naive T cells. However, by day 22 post-treatment, there were no differences between two groups in the frequency of naive and memory CD4+ T cells (Additional file 1: Figure S3). We did not find any differences between isotype IgG treated and non-treated NOD mice (data not shown).

### ATG therapy does not affect the response of the remaining non-depleted T cells to TCR stimulation, but induces decreased levels of Th1 and enhanced levels of IL-10-producing T cells

**Figure 3:**
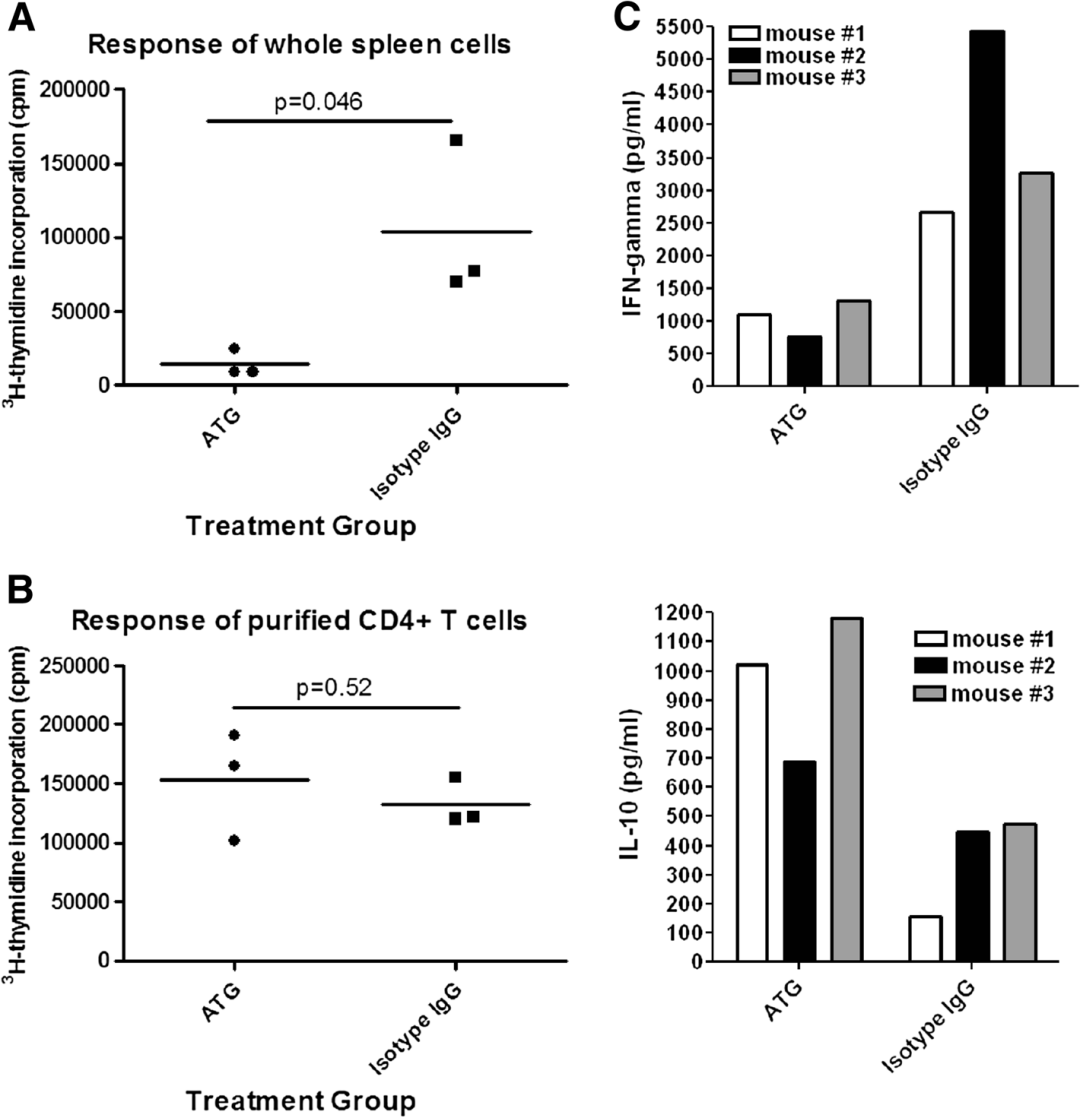
**Proliferation of T cells from mice treated with ATG or isotype IgG in response to CD3 antibody stimulation. **NOD mice were treated with ATG or isotype IgG twice with a 3-day interval. **A, **At day 3 post treatment, spleen cells were prepared and stimulated with anti-CD3 antibodies for 3 days, then ^3^H-thymidine (1 uCi/well) was added to the cultures for additional 16 hours. ^3^H-thymidine incorporation was measured by scintillation counting. Three mice were tested in each group. **B, **part of the above spleen cells was used to isolate CD4+ T cells. The purified CD4+ T cells were stimulated with anti-CD3 and anti-CD28 antibody coated beads for 3 days. Thereafter, ^3^H-thymidine (1 uCi/well) was added to the cultures for additional 16 hours. T cell proliferation was examined by the method described above. **C**, the production of IL-10, IFN-γ in the cultures from 3 animals in each group in **B **was measured by Luminex. The values for IL-10 and IFN-γ for treatment group and controls are (993.3±141.0 v.s. 366.7±96.1) and (1117.0±202.8 v.s. 3793±795.5), respectively. The difference between the two groups for IL-10 and IFN-γ by Student *t *test is significant with p value as of 0.0221 and 0.0253, respectively. An additional independent experiment was performed with the similar results.

To determine whether the immune response of T cells that are not depleted by ATG therapy alters, at day 3 post-treatment, we prepared spleen cells from ATG or isotype IgG treated NOD mice and stimulated them with anti-CD3 antibodies. T cell proliferation was measured by ^3^H-thymidine incorporation assay. We found that the proliferation of T cells from spleen cells of ATG-treated animals was significantly lower than those obtained from isotype IgG treated animals (Figure 3A). The reduced T cell proliferation was likely attributable to the lower frequency of T cells in spleen cells from ATG-treated mice. To address whether ATG treatment directly modulates T cells and alters their response to TCR activation, we purified CD4+ T cells (purity >95%) and stimulated them with CD3 and CD28 antibody coated Dynabeads. Then, T cell proliferation was examined by ^3^H-thymidine incorporation assay. We did not observe significant differences in terms of T cell proliferation between the two groups (Figure 3B). Characterization of cytokine-producing profiles of CD4+ T cells demonstrated that CD4+ T cells from ATG-treated mice produced significantly higher levels of IL-10 with reduced levels of IFN-γ, in contrast to those from isotype IgG treated mice (Figure 3C). These data indicate that ATG therapy skew T cell responses to IL-10-producing T cells including Th2 and Tr1.

### CD4+Foxp3+ Tregs are less sensitive to ATG depletion and remain at an increased frequency post CD4+ T cell recovery

**Figure 4:**
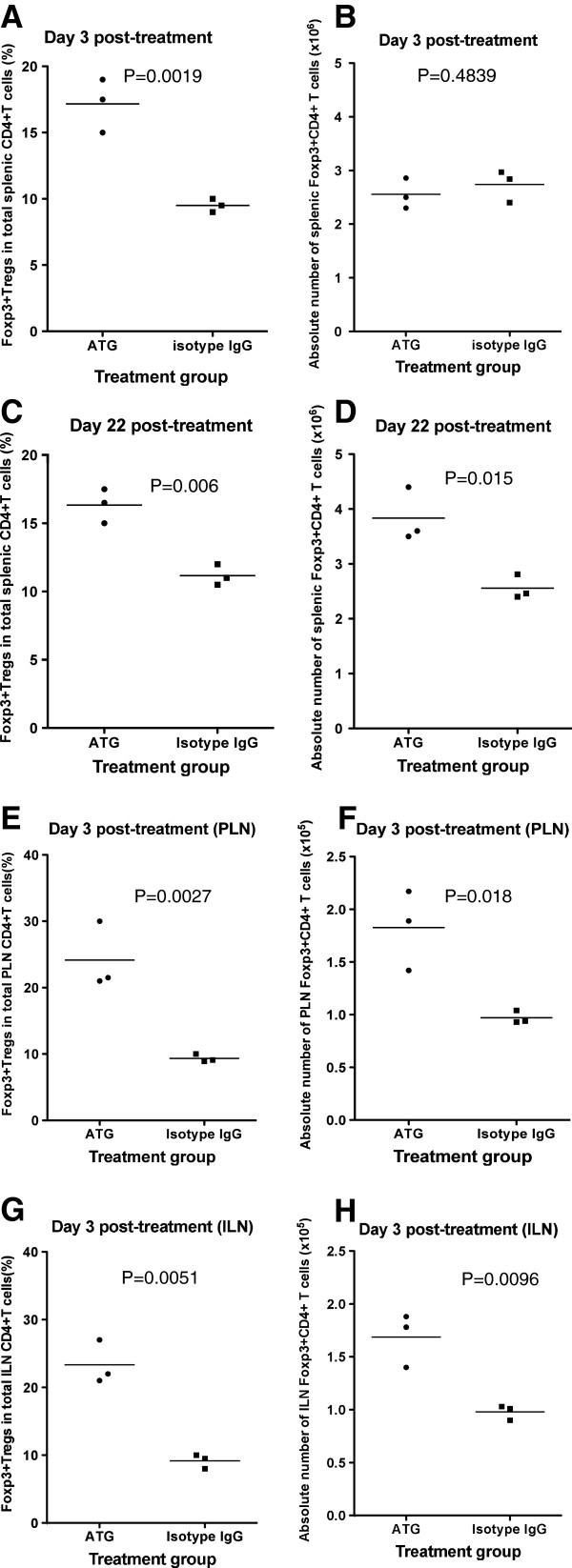
**Effect of ATG therapy on Foxp3+ Treg cells. **NOD mice were treated with ATG or isotype IgG twice with a 3-day interval. At day 3 and 22, CD4+Foxp3+ regulatory T cells in spleen were examined by flow cytometry and the summary of each group was shown in Figure 4A and B, and 4C and D, respectively. At day 3, CD4+Foxp3+ regulatory T cells in pancreatic and inguinal lymph nodes were also examined and shown in Figure 4E and F, 4G and H, respectively. Three mice were included in each group. The similar results were obtained in additional two independent experiments.

To determine whether Tregs undergo depletion post-ATG therapy, NOD mice at 6 weeks of age were treated with ATG or isotype IgG as described earlier. We then sacrificed one-half of the mice (n=3) from each group at day 3, and the rest (n=3) at day 22 post-treatment, respectively, and examined the proportion of Foxp3+ CD4+ T cells of total CD4+ T cells as well as their absolute numbers. As shown in Figure 4A and B, compared to the isotype IgG group, the ATG group demonstrated a significantly increased percentage of CD25+Foxp3+ CD4+ T cells in total CD4+ T cells at day 3 post-therapy and comparable levels of absolute numbers of these cells, suggesting that Foxp3+ Tregs were resistant to ATG mediated depletion. Moreover, at day 22, when CD4+ T cells were recovered, Tregs remained at higher levels in the ATG treated group than in the isotype IgG group (Figure 4C and D). Of interest, the proportionate increase of the Tregs out of total CD4+ T cells in lymph nodes was even more dramatic in the ATG compared to the isotype IgG group at day 3 post-treatment (Figure 4E and G). The same trend was also observed for Treg’s absolute numbers (Figure 4F and H).

### ATG therapy drives more CD62L- memory type Tregs

**Figure 5:**
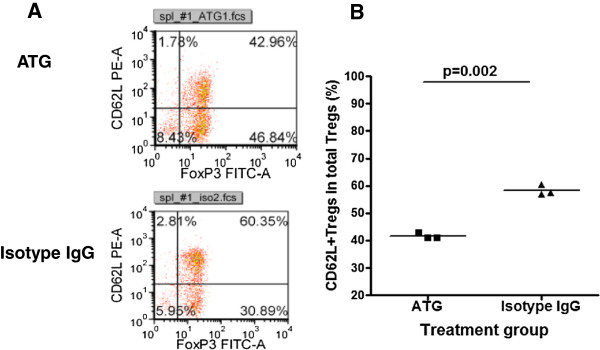
**Effect of ATG therapy on naive and memory Tregs. **NOD mice were treated with ATG or isotype IgG twice with a 3-day interval. At day 3 post treatment, the mice were sacrificed. The spleen cells were prepared and stained with CD4, CD25 CD62L and Foxp3 antibodies and examined by flow cytometry. CD62L+Foxp3+ and CD62L-Foxp3+ Tregs were analyzed by gating the CD4+CD25+ cell population as shown in Figure 5A. The summary of splenic CD62L+Tregs was shown in Figure 5B. ILN: inguinal lymph node; PLN: pancreatic lymph node. An additional experiment was performed with similar results.

We demonstrated (Figure 2) that ATG preferentially depletes CD62L+ naïve T cells. It was then of interest to learn whether ATG therapy depleted naïve and memory Tregs differently. In these experiments, we examined splenic Tregs at day 3 post-ATG or isotype IgG treatment using flow cytometry. We gated CD4+CD25+ T cells and then analyzed for the expression of Foxp3 and CD62L. Surprisingly, we noted that ATG therapy significantly reduced CD62L+ naïve Tregs compared to isotype IgG treated animals (Figure 5A and B). This finding suggests that the preservation of CD62L- memory Tregs may play an important role for ATG to modulate antigen-specific immune responses.

### ATG therapy fails to eliminate antigen primed T cell, but skews antigen-specific immune responses to Th2 and/or Tr1 cells

**Figure 6:**
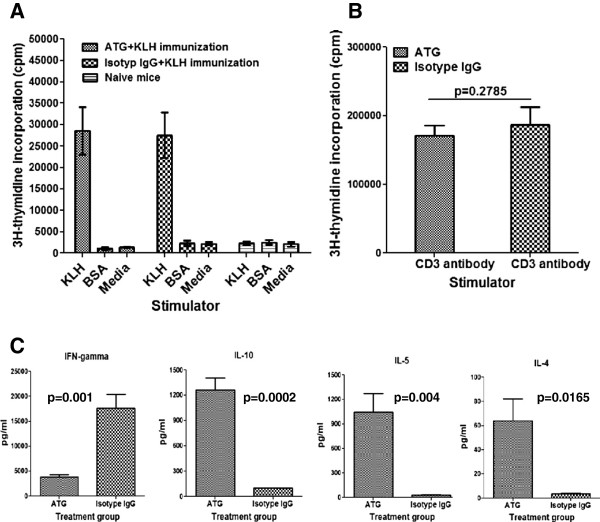
**Effect of ATG therapy on antigen-stimulated T cell proliferation and cytokine production. **NOD mice were treated with ATG or isotype IgG along with intraperitoneal injections of KLH in Alum as described in Methods. All mice were sacrificed at day 22 post treatment. **A, **spleen cells were incubated with KLH, BSA or media only; **B,** spleen cells were stimulated with anti-CD3 antibodies. All cultures were pulsed by ^3^H-thymidine (1 uCi/well) at day 4 for additional 16 hours. T cell proliferation was measured by scintillation counting. Triplicate wells were set up for each incubation. Three animals were included in each group. **C**, The production of cytokines in the cultures with KLH shown in Figure 6A was measured by Luminex assay. This experiment was repeated in additional two separate experiments with similar results.

As noted earlier, ATG has been used for a variety of immune-mediated conditions such as allogeneic transplantation and autoimmune diseases. One hope for ATG therapy would be its ability to eliminate most antigen-specific T cells so that immune responses against allograft or self-tissues could be avoided. For example, in settings of autoimmune diabetes, a majority of β cell antigen-specific T cells are presumably primed and become β antigen-specific effector or memory T cells. In allogeneic transplantation, alloantigen-specific T cells are activated to become effector or memory T cells unless there is immune intervention. To address how ATG therapy affects antigen-stimulated T cells, we designed and performed a series of experiments whereby NOD mice were treated with ATG or isotype IgG, along with simultaneous immunization by KLH protein in adjuvant alum. All mice were sacrificed at day 22 post-treatment, with spleen cells prepared and incubated with KLH, BSA (unrelated antigen) and medium alone. We found that KLH strongly stimulated spleen cells from both the ATG and isotype IgG treated mice to comparable levels (Figure 6A). Spleen cells from either group responded poorly to BSA stimulation (Figure 6A). These findings suggest that ATG therapy is unable to eliminate antigen-primed T cells. We also found that spleen cells from both groups responded to anti-CD3 antibody stimulation similarly (Figure 6B). However, we also observed that spleen cells from the ATG treated animals produced significantly increased levels of IL-4, IL-5 and IL-10 alongside of markedly reduced levels of IFN-γ in response to stimulation of KLH (Figure 6C), in comparison to isotype IgG treated animals. These findings suggest that despite the failure to eliminate antigen-specific T cells, ATG therapy induces antigen-specific T cell responses skewed to Th2 and Tr1, and largely limits the inflammatory Th1 response.

### ATG therapy fails to eliminate autoantigen-stimulated T cells

**Figure 7:**
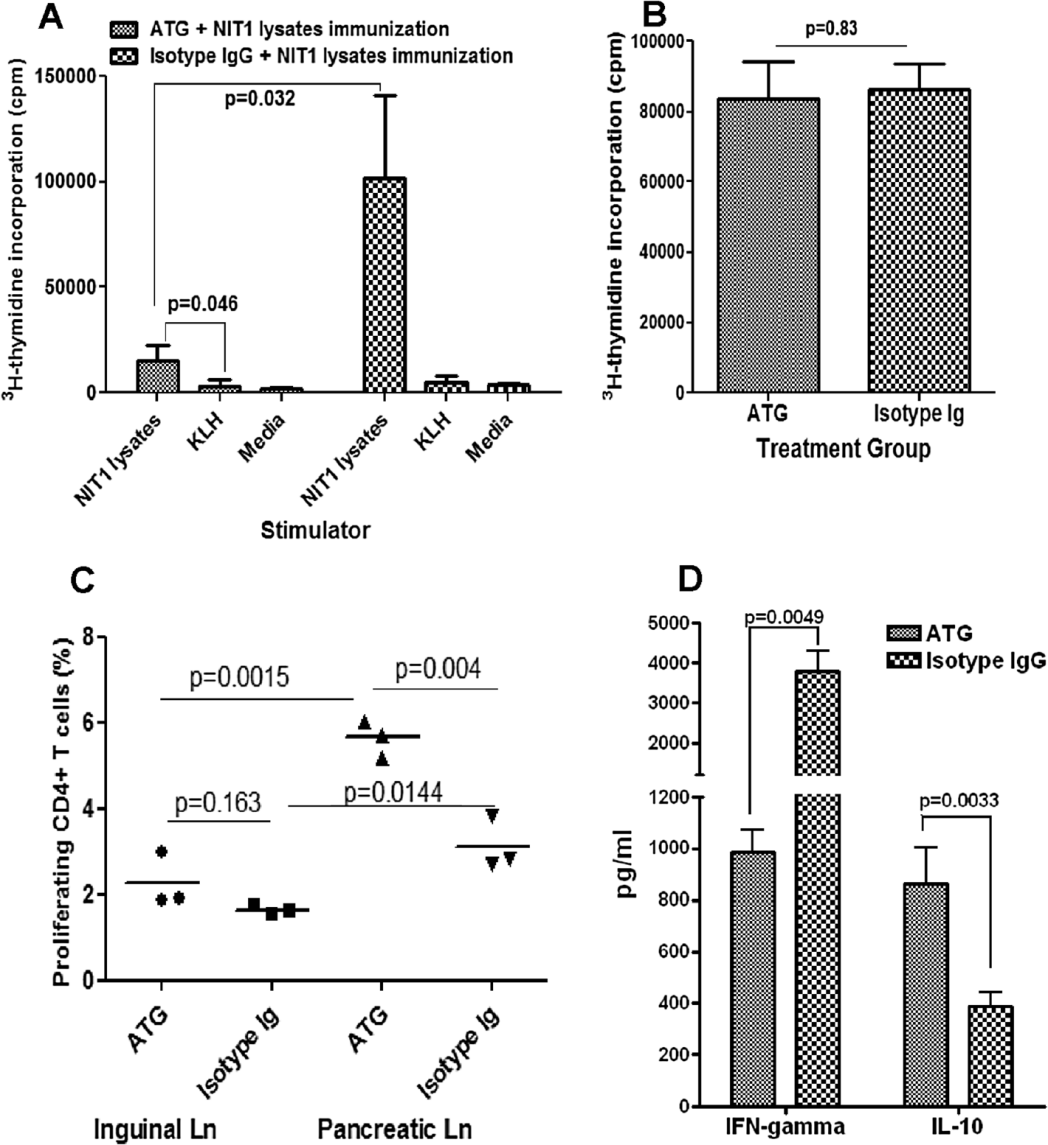
**Effect of ATG therapy on self antigen-specific T cells in vivo. A, **Eight weeks old NOD mice were treated with ATG or isotype IgG along with intraperitoneal injections of β cell antigens NIT1 lysates as described in the Method. A week post treatment, all mice were sacrificed and spleen cells were prepared. Spleen cells (1 × 10^6^/well) were incubated with NIT1 lysates, KLH (un-related antigen) or medium alone. Triplicate wells were set up for each incubation. T cell proliferation was measured by ^3^H-thymidine incorporation assay as described in the Methods section. Three mice were included in each group. Data shown represent the average of three mice+/−standard deviation (SD). **B,** Part of the spleen cells were used to isolate CD4+ T cells and the isolated CD4+ T cells (2 × 10^5^/well) were incubated with splenic dendritic cells (2 × 10^4^/well) purified from naive NOD mice in the presence of NIT1 lysates (20 μl/well). Triplicate wells were set up for each incubation. The proliferation of CD4+ T cells was measured by ^3^H-thymidine incorporation assay. Three mice were included in each group. Data shown represent the average of three mice+/−SD. **C,** Part of the mixed spleen cells from the above 3 ATG or 3 isotype IgG treated animals were stained with CSFE. Then, the CFSE-labeled spleen cells were intravenously injected into 8 weeks old naive NOD mice (3 mice per group). Four days later, the cells prepared from inguinal and pancreatic lymph nodes were stained with CD4-PerCp. The percentages of the proliferating CFSE-labeled CD4+ T cells (dilution of CFSE) were examined by flow cytometry. D, cytokines IFN-γ and IL-10 in the cultures shown in B were measured by luminex. Data shown represent average values of 3 mice+/−SD. An additional experiment was performed with similar results.

From the results above, it appeared that ATG therapy is unable to eliminate exogenous foreign antigen stimulated T cells (Figure 6A). It is of interest to know how ATG therapy affects self antigen-stimulated T cells. To address this issue, we stimulated β cell antigen-specific T cells in NOD mice at 6 weeks of age with an intraperitoneal injection of NIT1 (NOD mice insulinoma cell line) lysates along with ATG or isotype IgG treatment, as described previously. In these experiments, we did not wait until 22 days post treatment to assess T cell responses because of the concern that the exposure of endogenous β antigens would continuously prime T cells. Thus, at day 7 post-treatment, we sacrificed the mice, with the spleen cells prepared and stimulated with NIT1 lysates or unrelated antigen KLH. As expected, because of the transient significant depletion of T cells by ATG therapy, in response to stimulation of the NIT1 cell lysates, the proliferation of whole spleen cells from ATG-treated animals was significantly lower when compared to isotype IgG treated animals (Figure 7A). However, compared to the unrelated antigen KLH, NIT1 lysates still significantly stimulated spleen cells from the ATG treated animals (Figure 7A). To determine to what degree the ATG therapy depletes β antigen-specific T cells, we prepared spleen cells from the aforementioned mice and then performed an adoptive transfer of CFSE-labeled spleen cells to recipient NOD mice at 8 weeks of age. Four days later, CD4+ T cell proliferation of CFSE-labeled cells in inguinal and pancreatic lymph nodes was examined by flow cytometry. As shown in Figure 7C, in inguinal lymph nodes, the proliferation of CD4+ T cells of injected CFSE-spleen cells from ATG treated animals is slightly higher than that of CD4+ T cells from control group, which may reflect the higher T cell homeostatic proliferation potential post-ATG therapy [18]. More importantly, we found the proliferation of CFSE-labeled CD4+ T cells was much higher in pancreatic than in inguinal lymph nodes for both groups, which reflects β cell-antigen specific T cell response. However, unexpectedly, in response to endogenous β cell antigens, the proliferation of those injected CD4+ T cells from ATG treated animals in pancreatic lymph node was significantly higher than that of CD4+ T cells from isotype IgG treated animals. These results further confirm that ATG therapy fails to eliminate antigen-primed T cells. To confirm this, we demonstrated that purified CD4+ T cells from the spleen cells of both groups had comparable levels of proliferation in response to stimulation of syngenic splenic DC plus NIT-1 lysates (Figure 7B). Nevertheless, consistent with the results shown in Figure 6C, the cytokine assay for the cultures shown in Figure 7B demonstrated that ATG therapy suppressed β-cell antigen-specific IFN-γ production but enhanced IL-10 production by the CD4+ T cells (Figure 7D).

## Discussion

In this study, we questioned how ATG therapy affected naïve and memory T cells including Tregs with naïve or memory phenotypes. Until now, it was unclear to what extent ATG treatment affected naive and memory T cells pools. Resolving this issue is of great significance in managing ATG therapy in autoimmune diseases as well as in allogeneic transplantation. In line with the previous reports [12], we found that ATG therapy markedly depleted CD4+ and CD8+ T cells from the peripheral blood, and largely spared B cells and granulocytes. By day 3 post-treatment, T cell numbers reached their nadir. By day 22, CD4+ T cells recovered to within normal ranges, but CD8+ T cells remained lower than baseline. It has been suggested that ATG therapy may have differential effects in depleting T cells in peripheral blood and lymphoid organs dependent on dosing [19]. In this study, we tested our treatment protocol with optimal efficacy in preventing or reversing type 1 diabetes (500 μg/mouse × 2 doses 3 days apart) as described in our other reports [11,12]. To determine the efficiency of our ATG treatment protocol in depleting T cells from the lymphoid organs, we examined splenic CD4+ and CD8+ T cells in both groups (ATG versus isotype IgG) at day 3 post-ATG therapy. We found that the depletion of both CD4+ and CD8+ T cells was less efficient in spleen than from peripheral blood. The similar results were obtained in lymph nodes such as inguinal or pancreatic lymph nodes (data not shown). Of interest, it appears that ATG therapy preferentially depletes CD62L+ naive T cells from the blood because the proportion of CD62L+CD4+ naive T cells was markedly reduced while CD44+CD62L- CD4+ memory T cells as a fraction of total CD4+ T cells were increased. We observed a similar significant trend in the change of naive and memory T cells in spleen as well. Whether this differential depleting effect of ATG exhibits in local lymph nodes, especially in pancreatic lymph nodes is of interest to be further addressed. It is unlikely that the increase of memory T cells at day 3 post-ATG therapy is due to the conversion from naïve T cells [20,21] because ATG is still depleting T cells during this short period of time post-ATG therapy, and homeostatic proliferation unlikely leads to much in the way of T cell conversion.

The relative resistance of memory T cells to ATG-induced T cell depletion would allow for survival of memory T cells which potentially could lead to the recurrence of allograft rejection or autoimmunity after reconstitution of immune system post-ATG therapy. Consistent with this, we demonstrated that the proliferation of spleen cells from mice receiving ATG and de novo KLH immunization was as high as that of spleen cells from isotype IgG treated animals in KLH recall responses in vitro. We also found that β cell antigen-primed T cells during ATG therapy could survive ATG depletion as well. However, despite unaffected T cell proliferation in response to antigen stimulation post-ATG therapy, the T cell cytokine-producing profile in ATG treated animals indicated that ATG therapy skewed Th2 and possibly IL-10-producing Tr1, and reduced IFN-γ-producing Th1 responses. We had previously shown long-term reversal of diabetes in NOD mice using ATG or ATG in combination with G-CSF [11,12] and our current findings in this report provide a mechanistic basis for this in the skewing toward Th2 and/or IL-10-producing Tr1 responses under the regimen of ATG. In this study, although we focused our studies on CD4+ T cells, it is also important to study phenotypic and functional alterations of CD8+ T cells by the ATG therapy, given the pathogenic role of CD8+ T cells in type 1 diabetes, which will be addressed in the following future studies. The memory T cell phenotypic characteristics, as well as the functional alterations post-ATG therapy, may allow the modulated antigen-primed T cells to efficiently exert their regulatory functions in the periphery through affecting their migrating and homing capabilities, thereby preventing the recurrence of autoimmunity in autoimmune diseases and allogeneic rejection in allogeneic transplantation. These findings also implicate that ATG therapy plus antigen vaccination could lead to synergistic effect on induction of antigen-specific immune tolerance. Such information would be of great significance for developing antigen-based immunotherapeutic strategy for autoimmune diseases such as type 1 diabetes.

Prior to this effort, several mechanisms underlying ATG immune modulation have been proposed. A common belief is that ATG therapy works by T cell depletion through complement-mediated cell lysis and activation-induced cell death. However, another view regarding ATG therapy is that this agent exerts immunosuppressive function beyond that of simple T cell depletion [7,9]. ATG therapy may modulate immune response in vivo through inhibiting chemokine-driven T cell chemotaxis [22]. It may also influence the interaction between T cells and endothelial cells through modulating expression of adhesion molecules [22]. Our recent study showed that ATG therapy eliminated certain subset of dendritric cells and induced tolerogenic dendritic cells [10]. In addition, ATG therapy may facilitate tolerance induction through ATG-mediated apoptosis of T cells; because T cell apoptosis induced by anti-CD3 therapy was recently demonstrated to be associated with CD3 antibody therapy-induced immune tolerance [23]. The skewing of antigen-specific Th2 and IL-10-producing regulatory T cells (i.e., Tr1) by ATG therapy demonstrated in the current study suggests that the non-depleted antigen-responding T cells, instead of causing immune attack, may lead to antigen-specific restoration of immune tolerance, which implies that ATG works as immune modulator rather than immune suppressant.

As suggested previously, Foxp3+ Tregs may play a major role in preventing autoimmune diabetes during ATG therapy [11,12]. However, it is incompletely understood whether ATG therapy depletes Tregs differently than conventional T cells and how ATG affects the distribution of Tregs in different lymphoid tissues. It is also unclear whether ATG therapy affects naïve and memory Tregs differently. In the present study, we demonstrated that ATG therapy was less efficient in depleting CD4+Foxp3+Tregs and as a result, the proportion of CD4+Foxp3+ Tregs in CD4+ T cells was significantly increased in ATG treated animals compared to controls. This increase is even more dramatic in lymph nodes with greater than a doubling in the frequency of Tregs within total CD4+ T cells in ATG treated as compared to isotype IgG treated animals. In some animals, the percentage of Foxp3+ Tregs reaches 30% of total lymph node CD4+ T cells. Unlike equivalent absolute numbers of splenic Tregs in both groups, the absolute number of Tregs in lymph nodes was significantly higher in ATG than in Isotype IgG treated group at 3 days post treatment, suggesting that more Tregs were recruiting to the lymph nodes besides resistance to ATG depletion. The increase of Tregs in lymph nodes may be of great immunological significance for ATG to control local antigen-specific immune responses in the settings of autoimmunity such as type 1 diabetes, as well as in allogeneic transplantation. This increase of Tregs 3 days post-ATG therapy is unlikely due to the preferential proliferation of Tregs in the ATG-therapy induced lymphopenic animals [24] because the proliferation is limited in this short period of time especially still under active T cell depletion. Of interest, by day 22 post-ATG treatment when CD4+ T cells return to the normal levels, Tregs remained proportionately higher in the ATG group than in control group, which may be attributable to a faster proliferation of Tregs than conventional T cells [24] because Tregs possess superior capability to utilize IL-2 to conventional T cells [25]. This may also explain why a short-term ATG therapy offers a long-term protection in type 1 diabetes [11,12] and in allogeneic transplantation [2,26]. Intriguingly, Tregs with memory T cell phenotype were preferentially preserved in ATG therapy, which suggests that the preserved memory Tregs specific to certain antigens would be more potent in suppressing effector T cells reactive to the same antigens. As suggested recently [27], the memory Tregs may home to areas with active immunological reaction to quickly exert their regulatory function preferentially to naïve Tregs. This also explains the findings in our recent report that the post-therapy Tregs gain heightened immunosuppressive capacity [11]. There is evidence that the progression of autoimmunity in NOD mice leads to memory-like CD8+ Tregs which can be expanded in vivo by stimulation of nanoparticles coated with MHC-carried autoantigenic peptides. Of note, injection of these nanoparticles not only prevented T1D but also reversed overt diabetes in NOD mice [28]. Thus, the quantitative and qualitative changes of Tregs post ATG therapy may play an important role in suppressing antigen-specific effector T cells. Although Tregs are generally thought to suppress T cell responses in an antigen non-specific manner, emerging evidence shows that antigen-specific Tregs are more potent in suppressing antigen-specific T cell responses [29-31]. Lu, et al. reported recently that ATG therapy indeed induced self-antigen-specific Tregs in vivo that could provide long-term T1D protection in NOD mice [32]. Whether the increased memory Tregs post ATG therapy plus antigen challenge in our experimental settings contain more antigen-specific Tregs needs to be further explored.

## Conclusions

ATG therapy preferentially depletes naive T cells, largely spares Tregs and alters T cell cytokine-producing profiles. Thus, ATG therapy may modulate antigen-specific immune responses through inducing memory-like regulatory T cells as well as other protective T cells such as Th2 and IL-10-producing Tr1 cells.

## Competing interests

SE and JW are employees of Genzyme. Human Thymogobulin is a commercial product sold by Genzyme. Genzyme graciously supplied mouse thymoglobulin, but no financial support for the research described in this article. No other potential conflicts of interest relevant to this article were noted.

## Authors’ contribution

CQX designed the study, analyzed the data and wrote the manuscript; AVC performed the mouse in vivo experiments and data analyses, CHW, SW and BML performed flow cytometric assay and T cell proliferation assay; SE and JW provided ATG and discussed ATG-related issues; MJCS and MAA participated in the experimental design and helped draft the manuscript. All authors read and approved the final manuscript.

## Supplementary Material

Additional file 1 **Figure S1. **The effect of ATG therapy on absolute numbers of CD4+ and CD8+ T cells. **Figure S2. **The effect of ATG therapy on depleting spleen cells at day 3 after ATG injection. **Figure S3. **The proportions of naive and memory CD4+ T cells after CD4+ T cell number recovered from ATG therapy. Click here for file
