# Supplementary material for: Anti-thymocyte globulin (ATG) differentially depletes naïve and memory T cells and permits memory-type regulatory T cells in nonobese diabetic mice
Source: BMC Immunol. 2012 Dec 14;13:70. doi: 10.1186/1471-2172-13-70 (PMC3547787; doi:10.1186/1471-2172-13-70)
Supplement: Additional file 1 — Figure S1. The effect of ATG therapy on absolute numbers of CD4+ and CD8+ T cells. Figure S2. The effect of ATG therapy on depleting spleen cells at day 3 after ATG injection. Figure S3. The proportions of naive and memory CD4+ T cells after CD4+ T cell number recovered from ATG therapy. [file 1471-2172-13-70-S1.pdf]

Supplementary figure 1

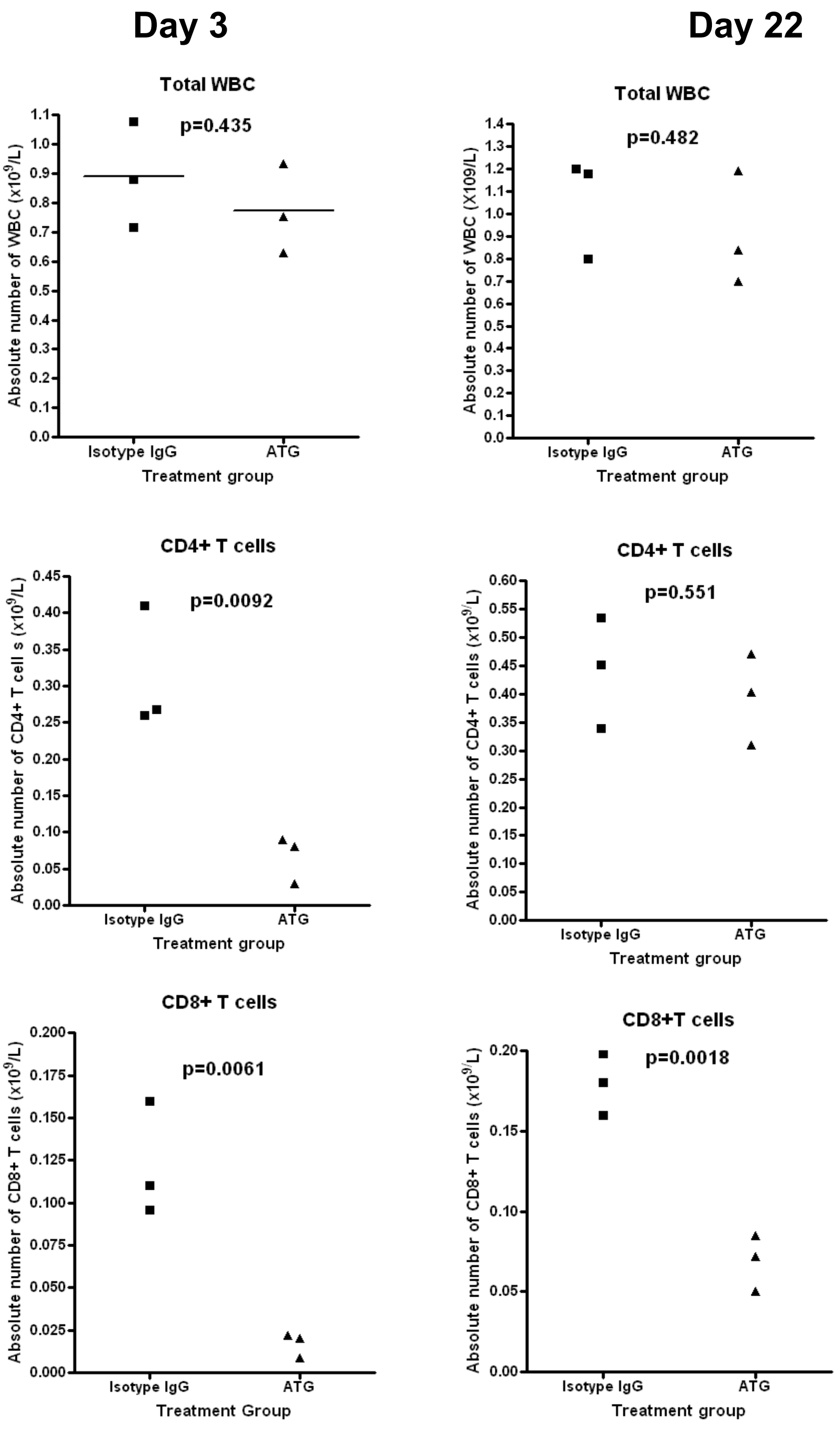

sFig 1. Absolute numbers of total blood white blood cells, CD4+ and CD8+ T cells post-ATG therapy. NOD mice were treated with ATG as described in the Method section. At day 3 and Day 22, the blood samples were collected. Peripheral blood white blood cells (WBC) were counted using hemacytometer. CD4+ and CD8+ T cells were calculated based on the total WBC numbers and the percentages of CD4+ T cells and CD8+ T cells in total WBC by flow cytometry. Three animals were included in each group.

Supplementary Figure 2

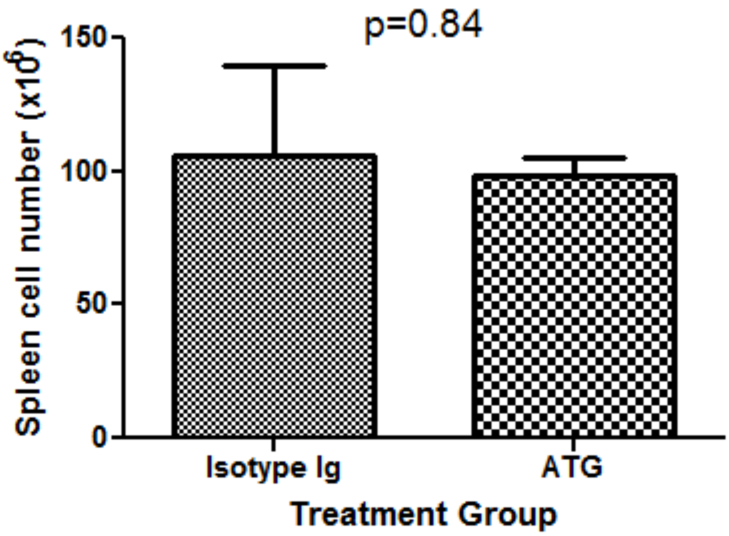

sFig 2. Total spleen cell numbers at day 3 post treatment. NOD mice were treated with ATG (n=3) and isotype Ig (n=3) as described in Method section. The mice were sacrificed at day 3 after the treatment started. The spleen cell numbers were counted by hemacytometer.

Supplementary Figure 3

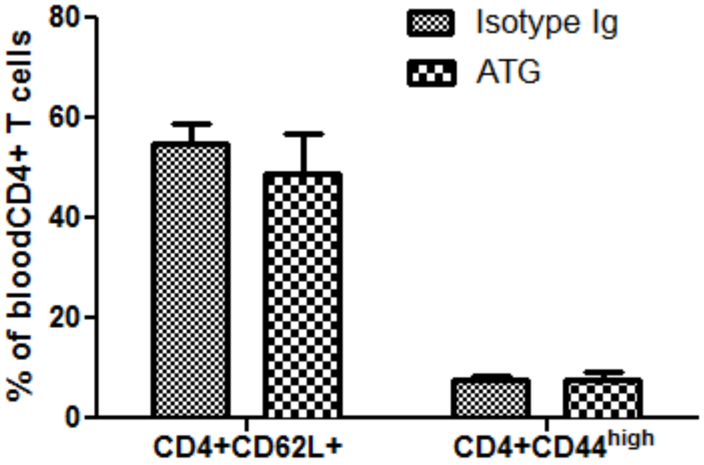

sFig 3. CD4+CD62L+ and CD4+CD44+ T cells at day 22 post-ATG therapy. NOD mice were treated with ATG (n=3) and isotype Ig (n=3) as described in Method section. CD4+CD62L+ and CD4+CD44+ T cells in peripheral blood total CD4+ T cells were analyzed by flow cytometry at day 22 after the treatment started.
